# Immunohistochemical Expression of Collagens in the Skin of Horses Treated with Leukocyte-Poor Platelet-Rich Plasma

**DOI:** 10.1155/2015/893485

**Published:** 2015-07-07

**Authors:** Maria Verônica de Souza, Mariana Brettas Silva, José de Oliveira Pinto, Marianna Barros de Souza Lima, Júlio Crepaldi, Gabriela Francine Martins Lopes, Hélio Batista dos Santos, Rosy Iara Maciel de Azambuja Ribeiro, Ralph Gruppi Thomé

**Affiliations:** ^1^Departamento de Veterinária, Universidade Federal de Viçosa, Campus Universitário, Avenida P.H. Rolfs, s/n, 36570-900 Viçosa, MG, Brazil; ^2^Clínica Médica Planeta Animal, Rua Araguaia 30, Bairro São Mateus, 36025-240 Juiz de Fora, MG, Brazil; ^3^Laboratório de Patologia Experimental, Universidade Federal de São João del-Rei, Campus Centro-Oeste Dona Lindu, Rua Sebastião Gonçalves Coelho 400, Bairro Chanadour, 35501-296 Divinópolis, MG, Brazil; ^4^Laboratório de Processamento de Tecido, Universidade Federal de São João del-Rei, Campus Centro-Oeste Dona Lindu, Rua Sebastião Gonçalves Coelho 400, Bairro Chanadour, 35501-296 Divinópolis, MG, Brazil

## Abstract

This study evaluated the immunohistochemical expression of type I (COL I) and III (COL III) collagens during the healing process of skin treated with leukocyte-poor platelet-rich plasma (LP-PRP). Seven healthy gelding crossbred horses aged 16 to 17 years were used. Two rectangle-shaped wounds were created surgically in the right and left gluteal regions. Twelve hours after wound induction, 0.5 mL of the LP-PRP was administered in each edge of the wounds of one of the gluteal regions. The contralateral region was used as control (CG). Three samples were obtained: after wound induction (T0), 14 days (T1) of healing process, and after complete closure of the skin (T2). The normal skin (T0) showed strong staining for type III and I collagen in papillary and reticular dermis, respectively. In the scar of the treated group, COL III showed important (*p* < 0.05) increase in immunoreaction in T2 compared with T1. The administration of a single dose of LP-PRP 12 h after induction of wound in horses does not influence formation of collagens I and III. However, the intense labeling for COL III suggests that the tissue was still weak during the macroscopic closure of the wound, demonstrating that healing was not completely finished.

## 1. Introduction

Large animals are considered excellent models for the study of wounds, given that they provide the proper comparison of the many physiological and immunological aspects involved in wound healing [1]. Different cell types, cytokines, and extracellular matrix molecules at the wound site interact with different systemic factors such as platelets, the coagulation cascade, and humoral cell components, which together enable the healing of wounds [2].

The most appropriate form of healing a cutaneous wound is by first intention [3, 4], but this procedure is limited to wounds located in anatomical regions that allow for the excision and adaptation of its edges [3]. Healing by second intention, on the other hand, which involves two independent processes—contraction and reepithelialization [5]—is often the only possibility to close wide and deep wounds. However, the process is slow and may result in the formation of exuberant granulation tissue. In this sense, new therapies aiming to reduce the maximum healing period of cutaneous wounds and that result in the formation of a tissue as similar as possible to a healthy one emerge every day.

Platelet-rich plasma (PRP), a product derived from the whole blood centrifugation, is an autogenous and economic source of a variety of growth factors that participate actively in the healing process of cutaneous wounds such as the transforming growth factor beta (TGF-*β*), because it is associated with the stimulus to the synthesis of collagen [6–8] both type I and type III, which are the main structural components of the mature scar tissue, and it is synthesized by fibroblasts [9, 10].

Despite the numerous studies conducted with platelet-rich components in different tissues and in various species, there is still not enough evidence confirming the effectiveness of PRP in the treatment of cutaneous wounds. This is partly due to the region of the body subjected to the treatment; the form of obtaining and the composition of the PRP (considering the amount of platelets and leukocytes); its physical form (liquid/gel), route, and frequency of administration; volume; and the moment it is administered during the skin healing process; among other aspects. There are some studies and case reports on horse skin [11–16], but with contradictory results. Moreover,* in vivo* research studies evaluating the therapeutic response using highly specific and sensitive methods are rare. According to Furness et al. [17], one of the most indicated techniques to identify the collagens is immunohistochemistry, which may delineate the type present in the evaluated tissue. This methodology has been used to determine the expression of growth factors such as TGF-*β*1, which is the most often studied isoform in the different species, because in addition to participating in the synthesis of collagens (as previously mentioned) it is essential for angiogenesis, chemotaxis, and cell proliferation [18].

Considering the amount of platelets and leukocytes, the platelet concentrates can be classified into different categories. There is the pure PRP (P-PRP), where the white cells are removed intentionally, and the PRP with leukocytes (L-PRP) [19]. There are also the components rich in fibrin (PRF) [20] that, unlike the other concentrated, do not require the use of anticoagulants to be obtained [21]. L-PRP should have between 5x and 8x more platelets [19, 20] and three times or more amounts of leukocytes [20] than the whole blood. However, there is no consensus of the amount of platelets that the PRP should present for it to be regarded as pure. Hutchins and Grabsch [22] report that it should contain a moderate number of platelets, which should be 1.5x to 2.5x the existing in whole blood, while Carmona et al. [20] consider that must have 1.3x to 4x the blood concentration. These authors also mention that the concentrate can contain 0.5x to 2x more leukocytes than the blood. When the concentrate rich in platelets is activated by pharmacological agents, forming a fibrin polymer, it is known as platelet gel (PG). Therefore, a PG obtained from a P-PRP is called P-PRG, and when it is originated from L-PRP, it is known as L-PRG. Finally, taking into account the basic definitions for those components rich in platelets, there is also the PRP poor in leukocytes (LP-PRP). This last component may results in lesser acute inflammatory response, lower cellularity, and vascularity when compared to PRP with a high amount in white cells (LR-PRP), as reported by Dragoo et al. [23] five days after treatment of tendon of rats with LP-PRP or LR-PRP.

In physiological conditions, the repair process requires activation and/or suppression of several substances that may result in abnormal healing of the cutaneous wound. The immunohistochemical evaluation of proteins expressed in the different healing phases of PRP-treated skin may provide invaluable information to validate the effectiveness or ineffectiveness of the therapy. Thus, the objective of the present study was to evaluate the immunohistochemical expression of type I and III collagens during different phases of the macroscopic healing process of skin treated or untreated with LP-PRP.

## 2. Material and Methods

This research was approved by the Ethics Committee on Animal Use of Universidade Federal de Viçosa (UFV) (protocol number 35/2013). The procedures were conducted according to the Rules of Conduct for the Use of Animals in Teaching, Research and Extension of the Department of Veterinary (DVT/UFV), the Medical Veterinary Professional Ethics Code, the Ethical Principles for Animal Research established by the Brazilian College for Animal Experimentation (COBEA), and the current Brazilian Legislation.

Seven healthy gelding crossbred horses aged 16 to 17 (16.37 ± 0.52) years were used. Only seemingly healthy animals with no dermatological disorders were included in the study. The animals were housed in individual 20-m^2^ stalls fifteen days prior to the onset of the experiment, where they were fed Tifton 85 hay and chopped elephant grass (*Pennisetum purpureum*), in addition to a mash diet for horses. Mineral salt and water were provided* ad libitum*. This management was carried on over the course of the experimental trial. In the period of adaptation the horses were weighed, bathed with an acaricide solution containing deltamethrin, and dewormed orally with moxidectin paste (0.2 mg/kg). Stalls were cleaned twice daily to remove the excreta and replace the bedding.

Three rectangle-shaped skin injuries were created in the right and left gluteal regions of all animals, as described by Ferreira et al. [24]. For this purpose, the sites were previously clipped and aseptically prepared with 2% germicide chlorhexidine digluconate and 0.5% alcoholic solution. The animals were injected intravenously with 2% xylazine (0.8 mg/kg) and then subcutaneous nerve-block was performed with 2% lidocaine hydrochloride around the site to be incised, using a 21-gauge needle.

To surgically create the wounds, a scalpel and a 2.5 cm sided (6.25 m^2^) rectangle-shaped plastic mold were used to remove the skin fragment (epidermis, dermis, and subcutaneous tissue). Wounds were identified as A and B, from cranial to caudal on both gluteal region. The first collection was considered time zero (T0). The wounds healed by second intention and were monitored until complete closure, when the last biopsy was performed (wound B). During the postoperative period the surgical wound was dressed daily with gauze soaked in Milli-Q water. All animals received antitetanus serum on the day of the injury. Their pain was lessened by using a single intravenous dose of butorphanol tartrate (0.08 mg/kg). No anti-inflammatory or antibiotic was used, as they could hamper or make it impossible to evaluate the treatment with LP-PRP.

The leukocyte-poor platelet-rich plasma was obtained by following the procedures described by Argüelles et al. [25]. For this purpose, 144 mL blood samples were collected from each animal, by puncturing the external jugular vein in 36 Vacutainer tubes with 3.2% sodium citrate (0.199 mol/L). The tube capacity was 5 mL : 0.5 mL for sodium citrate and 4.5 mL for whole blood. Blood samples with EDTA were also collected to quantify platelets and total leukocytes.

The samples of blood to obtain the LP-PRP were homogenized and centrifuged at 120 ×g for ten minutes. After this first centrifugation, 50% of blood plasma from the surface was discarded and the rest was transferred to four polypropylene tubes with 10 mL capacity without anticoagulant. Leukocyte button and sedimented erythrocytes were discarded. Next, the plasma was centrifuged one more time at 240 ×g for 10 min. After this second centrifugation, the plasma was divided into two fractions: the supernatant (platelet-poor plasma) and the remaining fraction, named platelet-rich plasma. A volume of 75% of the obtained plasma present at the surface of each tube was discarded and the PRP containing the platelet button was reserved.

The concentration of platelets and leukocytes was determined manually [26] in the LP-PRP and the blood obtained with EDTA. The count was performed in a Neubauer chamber using Türk's solution to count the leukocytes and Brecher's method for platelet count [27].

All the horses were subjected to a local treatment with LP-PRP 12 h after the injuries were performed. After clipping and sedation with 2% xylazine intravenously (0.8 mg/kg), one of the gluteal regions—chosen randomly—was prepared aseptically for the administration of the LP-PRP (treated group = TG). The wounds in the contralateral gluteal region (control group = CG) did not receive any infiltration, but only local cleaning with Milli-Q water, as was done in the treated wounds.

**Figure 1:**
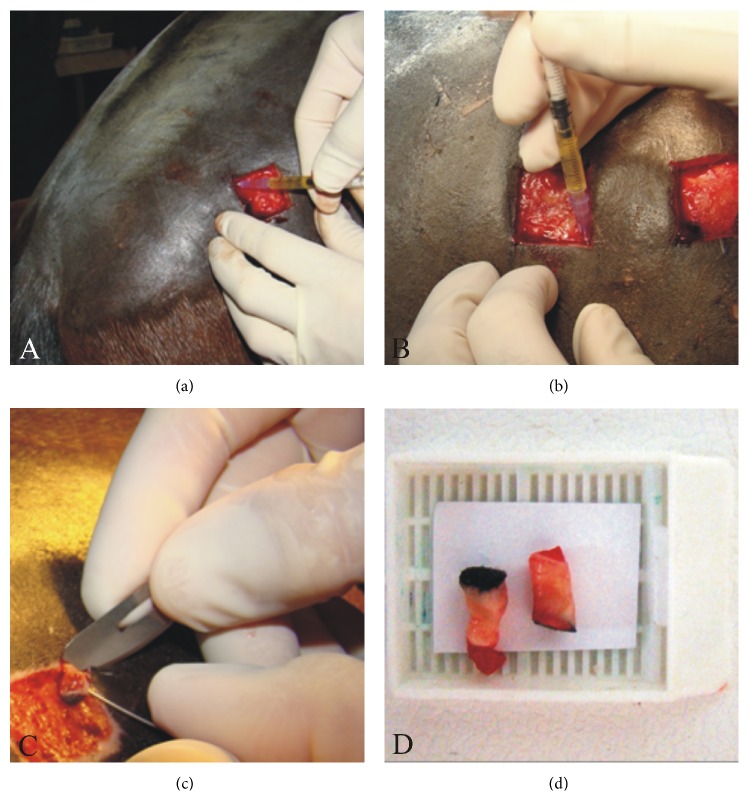
Administration of 0.5 mL leukocyte-poor platelet-rich plasma on the wound edges, using a 24-gauge needle (a, b). Skin sample collection using a scalpel and a needle, after cutting with a 6 mm diameter* Punch* (c). The obtained fragments were immediately placed on histology cassettes (d) and fixed in 10% formalin.

The platelet-rich plasma was prepared immediately before its administration, which was performed using a 24-gauge needle. Each edge of the two wounds (A and B) received 0.5 mL of the LP-PRP (Figures 1(a)-1(b)), totaling 2 mL per wound and 4 mL per side (left or right) of the gluteal region. After the treatment, the animals were kept in stalls and let out for a daily period of 2 h and monitored throughout the experiment.

Skin samples were collected to analyze the immunohistochemical expressions of type I and III collagens. Skin fragments were obtained by biopsy (full thickness) using a 6 mm diameter* Punch* with the aid of a scalpel at 14 days (T1) after wound A. In addition, a new collection was made when wound B was completely closed (T2).

For the biopsies, the animals were sedated intravenously with 2% xylazine (0.8 mg/kg) and then subcutaneous block anesthesia was performed with 2% lidocaine hydrochloride without vasoconstrictor around the site to be biopsied, using a 21-gauge needle. The last collection (T2) was performed in the center of the healed area (which was still unpigmented), whereas the second biopsy (T1) was obtained from the peripheral region of the wounds (Figure 1(c)), comprising both intact and wounded skin, including migration or reepithelialization, and granulation tissue, according to the collection time. Skin samples (Figure 1(d)) were fixed in 10% formalin, embedded in paraffin, sectioned with 5 *μ*m thickness, and stained with hematoxylin and eosin (HE) (Erviegas Instrumental Cirúrgico Ltda., São Paulo, São Paulo, Brazil**)** for an overall tissue-character assessment at the different times of sample collection. This analysis was conducted by three observers blinded to origin of the biopsy specimens.

Sections were also evaluated by immunohistochemistry using the indirect immunoperoxidase technique. The primary antibodies used are listed in Table 1. For the antigen retrieval, a pretreatment was performed with citrate buffer pH 6.0 at 95°C for 30 min in water bath. After washing with PBS, a 16% milk powder solution was used to block the nonspecific reactions and tissue permeabilization, and 3% H_2_O_2_ (Dinâmica 1857, Blueskylab Artigos para Laboratório, Guarulhos, SP, Brazil) during 30 min to block endogenous peroxidase. The sections were incubated with primary antibody overnight at 4°C. Subsequently, the histological sections were incubated with anti-mouse and anti-rabbit universal immunoperoxidase polymer (Histofine-Simple Stain MAX PO MULTI, Biogen Comercial e Distribuidora Ltda., Sumarezinho, SP, Brazil) and the immunohistochemistry reaction was revealed with DAB (DAKO-K346811-2, Labscience de Minas, Instrumentos Científicos Ltda., Belo Horizonte, MG, Brazil). Horse and rat tendons, pig skin, and mouse and human testicles were used for the positive control. For negative control, one of the slides did not receive primary antibody. The sections were counterstained with Harris hematoxylin (Labscience de Minas Instrumentos Científicos Ltda., Belo Horizonte, MG, Brazil). Images of the slides were acquired using an optical microscope and the Axio Vision software 4.8 Rel.

The immunostained area was evaluated in the healing area where the recently formed skin showed a thick stratified epithelium and dermis without presence of annexes at the different wound evolution times. The immunohistochemically stained area (IHS_*A*_) was calculated for collagen I and III primary antibodies as a percentage of the total area evaluated through the color segmentation analysis using software AxioVision 4.8.2. from Zeiss (http://www.zeiss.com.br). The brown stain was selected and a mask was subsequently applied to make a separation of permanent colors. The mean IHS_*A*_ was calculated from 30 images obtained in quadrant shape, starting from the underlying epithelial (papillary dermis) in the healing area toward the reticular dermis and then returning to the epithelium, for the time interval of treatment (IHS_*A*_ = 100*S*_*A*_/*W*_*A*_, where *S*_*A*_ = stained area and *W*_*A*_ = whole area).

The statistical analysis was conducted on GraphPad InStat version 3.05 (GraphPad Software, Inc.). The *t* test was utilized for independent samples to compare the control and treated groups at each time (T0, T1, and T2). In addition, the different times were compared with the initial situation (time 0) in both groups using the *t* test for paired data. Additionally, Student's *t*-test for independent samples was adopted to compare the mean values of platelet and leukocyte counts in the blood and LP-PRP and the mean wound-healing time in the groups. To compare the different times in both groups, an analysis of variance (ANOVA) of repeated measures was performed. All analyses were carried out at 5% significance. All data were expressed as mean ± SEM.

## 3. Results and Discussion

### 3.1. Macroscopic Wound Closure, Platelets and Leukocyte Counts

The period for the closure of a cutaneous wound depends on the extent and anatomic location of the injury, on the presence of local infections, among other aspects. According to Theoret et al. [4], the wound healing process in horses is very similar to that of humans. The skin surface is reepithelized, with the dermis healing by stromal granulation, migration of myofibroblasts, and wound contraction [28]. The skin healing process is classically divided into four well-defined overlapping phases, including haemostasis, inflammation, proliferation, and lastly remodeling [5, 29]. Schultz et al. [30] subdivide the third phase into migration and proliferation and the fourth into contraction and remodeling.

In the present study the maximum time necessary to heal the wounds was 47 days in both groups of animals, averaging 36.85 ± 7.45 days in the wounds that were not treated with LP-PRP, and 38.85 ± 6.46 days in the treated wounds; however, there was no difference (*p* = 0.59) between groups. Therefore, 37 days was considered as the mean time necessary for wound closure. This average healing time is close to that found in uncontaminated wounds located in the thoracic region, which is approximately four weeks [7], and of wounds performed in the same manner and in the same region as that described in the present study by Ferreira et al. [24] (38.5 ± 3.9 days). The obtained results are similar to those described by Monteiro et al. [13], who also observed that the maximum closure period of PRP-treated wounds was longer than that of untreated wounds, which suggests that the therapy has no positive effect on the maximum macroscopic healing time of the skin wound.

In the whole blood and LP-PRP the platelet count varied from 100,000 to 150,000 platelets/*μ*L and from 320,000 to 390,000 platelets/*μ*L, respectively. The leukocyte (white blood cells) values, however, varied from 5,600 to 10,900 cells/*μ*L and 50 to 900 cells/*μ*L in the whole blood and LP-PRP, respectively. The average number of platelets found in the blood was lower (*p* = 0.000) than in LP-PRP. The opposite (*p* = 0.000) occurred with the average leukocyte count.

The concentration of platelets in the LP-PRP was considered adequate, because it was above the number considered appropriate by Anitua et al. [31] and similar to that obtained in studies conducted with healthy crossbred horses [32–34]. Additionally, an* in vitro* study carried out by Graziani et al. [35] demonstrated that PRP preparations have a dose-specific effect on the proliferation of fibroblasts and osteoblasts. Better effects (considered optimal) were observed when the platelet count was 2.5x higher than that present in the whole blood. In contrast, higher amounts (5.5x) resulted in undesirable effects such as reduction of the fibroblastic proliferation and of the osteoblast function. According to the authors, different concentrations of platelets in the PRP can also lead to different results in* in vivo* studies.

The number of leukocytes (50–900 cells/*μ*L) present in the LP-PRP was also considered adequate, because it was lower than that obtained by Vendruscolo et al. [33] (2, 460 ± 763 cells/*μ*L), who utilized a relative centrifugal force (*g*) and the same time used in the present study. The positive effect of leukocytes on inflammation, immunity, and cell signaling is unquestionable [36], just as on the release of growth factors. On the contrary, undesirable effects are also attributed to the leukocytes, inasmuch as in their signaling proinflammatory cells they may cause tissue catabolism [37]. Moreover, there are reports that the application of platelet concentrate with leukocyte for the treatment of patients with tendinopathy may increase the local pain [29]. Therefore, both positive and negative effects are associated with the presence of these cells in the PRP. The use of a PRP with a low number of leukocytes, as was the case of the current study, is considered appropriate when aiming to promote a rather anabolic than catabolic action, given that the wounds were not contaminated.

### 3.2. Histological Evaluation of the Tissue Character of the Healing Process

**Figure 2:**
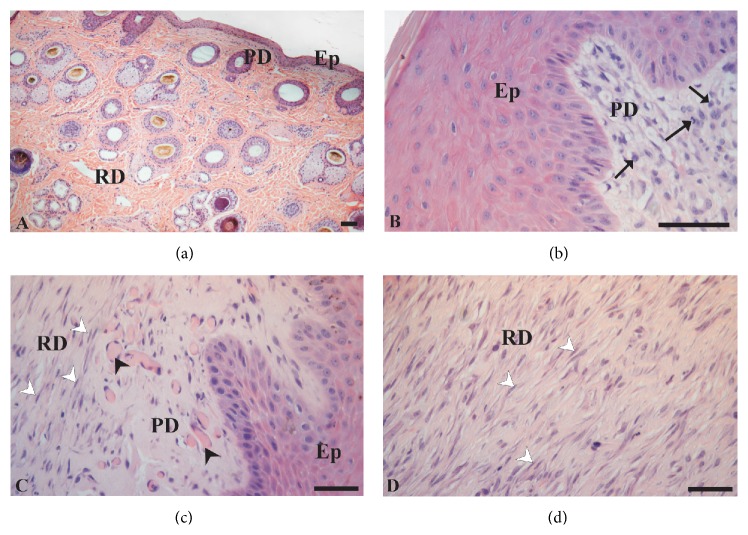
Photomicrography of skin before (a) and during the healing process (b–d), where (b) is after two weeks, and (c) and (d) after complete wound closure. Stratified squamous epithelial tissue (Ep), papillary dermis (PD), reticular dermis (RD), dilated capillaries (black arrowhead), fibroblasts (white arrowhead), and immune cells (arrows). HE stain. Bars = 50 *μ*m.

The images obtained in the histological analysis of the HE-stained skin are shown in Figure 2. At time zero (making of the wound) it was revealed that the skin had a normal character, that is, an epidermis with poorly keratinized, stratified squamous epithelial tissue, and dermis with two well-defined layers: papillary (superficial) and reticular (deep). Annexes hair follicle, hair erector muscle, sebaceous gland, and sweat glands were observed in these layers (Figure 2(a)).

The papillary dermis showed loose connective tissue with numerous capillary loops in contact with the basal layer of the epidermis. In addition, nuclei of connective tissue cells such as fibroblasts and fibrocytes were observed in this layer. The extracellular matrix has an amorphous ground substance in moderate quantity and thin but abundant collagen fibers. The reticular dermis was characterized by the presence of connective tissue cells, thick acidophilus collagen fibers arranged in various directions, and scarce amorphous ground substance. The adenomers and ducts of glands were more present in this layer.

The analysis of the animals' skin at two weeks showed that the surface of the wound was coated by dehydrated serocellular crust. Granulation tissue exhibited reduction in the number of vessels mainly in the deeper layers of the wound. Extracellular matrix showed predominance of thick collagen fibers, but still with poor organization. Fibroblasts showed round nuclei and acquired appearance of myofibroblasts in the deep dermis. Reepithelialization reached two thirds of the wound surface. There was a reduction in the inflammatory process (Figure 2(c)), but with a greater number of eosinophils in the wound in the LP-PRP-treated group.

Regardless of the group, the microscopic analysis performed at macroscopic wound closure, which as previously mentioned occurred approximately at 37 days, showed complete reepithelialization of the wound with hypertrophy and hyperplasia of the covering epithelium and remodeling of scar tissue. Fibroblasts show spindle-shaped nuclei and are organized into parallel bundles, acquiring a myofibroblast aspect. Areas of inflammation characterized by dilated capillaries (Figure 2(b)), recruitment of leukocytes, and particularly neutrophils were observed in the papillary layer. In addition, plasmocytes were also identified in the same layer. Better organization of fibroblasts with less deposition of collagen, and increased numbers of eosinophils were identified in the treated group compared with the control group. The reticular dermis in the wound healing area had a connective tissue arrangement similar to the dense ordered with fibroblasts aligned in parallel (Figure 2(d)).

The histopathological findings in the present study are compatible with the healing process described by Schwartz et al. [7], although they may vary depending on the region under evaluation. The authors studied the healing process using subjective histological evaluation of second-intention healing process of wounds from the thoracic region and the distal aspect of horse forelimbs by the Picrosirius Red Histochemical technique. The injuries located in the limbs had, at time zero, normal tissue with hair follicles, blood vessels, collagen, and few red cells. The inflammation was evident in the four weeks of assessment. In the second week, the predominant cells were particularly macrophages, and there were few spindle-shaped fibroblasts, and in the fourth week there was an increase in the collagen content and reduction of inflammatory cells. Wounds located in the thoracic region had dense collagen in all layers. After 14 days, superficial collagen deposition was observed, and the density of the collagen was increased throughout the depth of the tissues, and the fourth week showed collagen organized in parallel fibrils in all tissue layers. On the other hand, in the wounds located in the forelimbs, collagen was abundant in all tissue layers at time zero and increased gradually from week 1 to 4. However, the collagen fibrils were not aligned in an organized pattern compared with the samples obtained from the thoracic wounds. With respect to myofibroblasts, these cells were more organized in the wounds of the thoracic as compared with those of the limb in the third and fourth weeks of the healing process. In contrast, in the first two weeks there was no difference between the wounds.

According to Deschene et al. [38] skin biopsy samples should contain the skin in all its thickness, since keratinocytes should predominate in the epidermis of intact skin, whereas fibroblasts should reflect the depth of the dermis. During the early phase of skin-wound healing, inflammatory cells must prevail, with a subsequent reduction in the later stage. At this stage endothelial cells and fibroblasts must be present and the number of keratinocytes should increase for reepithelialization to take place. In the present study, although skin closure was earlier (*p* > 0.05) in the control group, the overall histological assessment revealed that fragments obtained in the final stage of the healing process of LP-PRP-treated wounds show a better organization of the collagen fiber bundles microscopically as compared with untreated wounds. Therefore, the macroscopic closure of the wound does not necessarily reflect the quality of the scar tissue.

This faster closure may be related to the process of physiologic repair of skin wounds, which classically occurs in four well-defined overlapping phases [5, 29, 30]. Although the amount of platelets present in the hematoma formed after a wound is about 4%, the remainder being erythrocytes (94%) and leukocytes (less than 1%) [39], growth factors such as TGF-*β* and platelet-derived growth-factor (PDGF) are released by the platelet *α*-granules at the site where there is tissue injury [40]. According to Marx [41], growth factors are actively secreted during the first 10 minutes after clotting.

Myofibroblasts, contractile cells that originate from the differentiation of fibroblasts, observed during the last phase of the skin healing process, are essential for wound contraction, enabling faster reduction of the wound area [42]. The process occurs faster than reepithelialization itself [43]. However, its persistence suggests that wound healing is still in progress [7]. In the present study the cells were observed in the evaluation of the already clinically healed skin.

### 3.3. Immunohistochemistry for Collagen Types I and III

**Figure 3:**
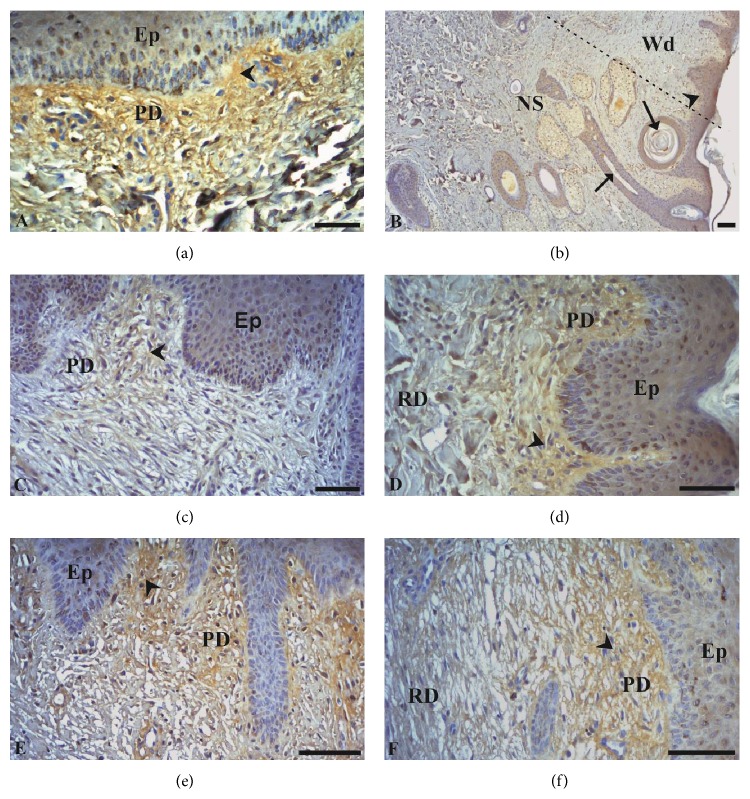
Immunohistochemistry for type III collagen in skin of horses treated or untreated with LP-PRP. Immunostaining was visible in the papillary dermis at all times and in all groups: normal skin (a), border tissue and scar area (b). Image obtained at 14 and 37 days of skin healing process from treated wounds (c and e, resp.) and controls (d and f, resp.). Stratified squamous epithelial tissue (Ep), papillary dermis (PD), reticular dermis (RD), immunostaining (arrowhead), hair follicle (arrows), scar edge skin (NS), and scar area (Wd). Bars = 50 *μ*m.

**Figure 4:**
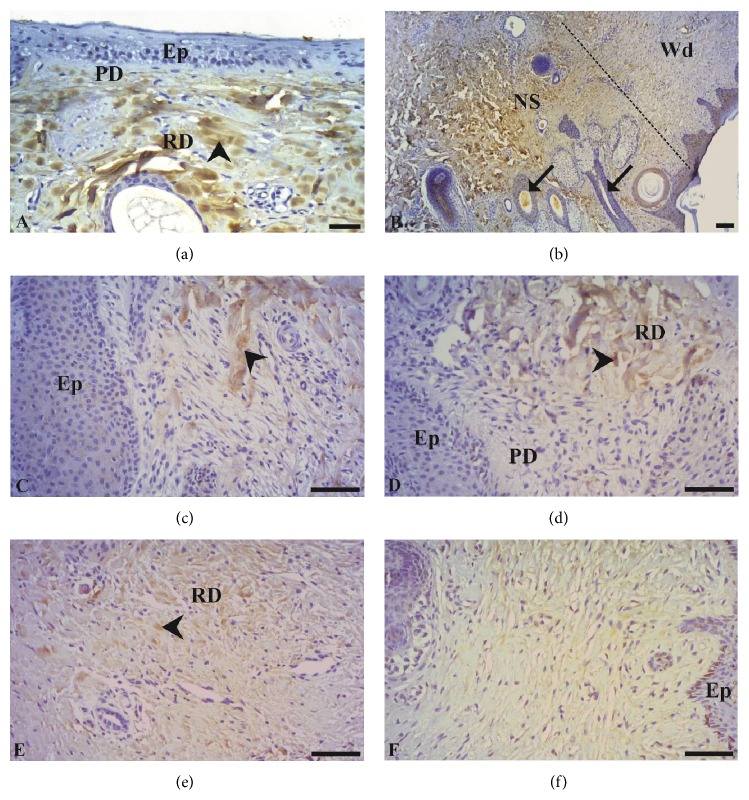
Immunohistochemistry for type I collagen in the skin of horses treated or untreated with LP-PRP. Immunostaining was visible in the reticular dermis at all times and in all groups: normal skin (a), border tissue and scar area (b). Image obtained at 14 and 37 days of skin healing process from treated wounds (c and e, resp.) and controls (d and f, resp.). Stratified squamous epithelial tissue (Ep), papillary dermis (PD), reticular dermis (RD), immunostaining (arrowhead), hair follicle (arrows), scar edge skin (NS), and scar area (Wd). Bars = 50 *μ*m.

Figures 3 and 4 show images obtained from the samples evaluated by immunohistochemistry. For COL III, staining in healthy skin (T0) showed reticular fibers located in the papillary dermis (Figure 3(a)). In contrast, for COL I, staining was evident in the reticular dermis (Figure 4(a)). The tissue of the edge of the scar had the same staining pattern as T0 in the papillary and reticular layers, with intense staining for COL III (Figure 3(b)) and I (Figure 4(b)), respectively. As previously mentioned, the immunostained area for COL III (Figures 3(c)–3(f)) and COL I (Figures 4(c)–4(f)) was evaluated in the healing area where the newly formed skin had thick stratified epithelium and dermis without annexes for the different wound-healing periods.

**Figure 5:**
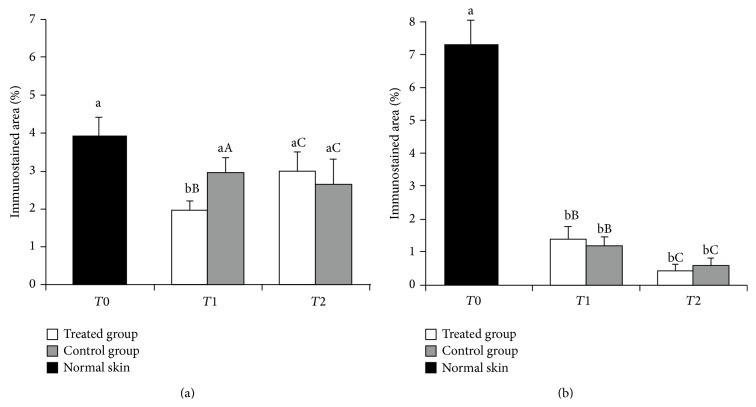
Percentage of immunostained area for collagen types III (a) and I (b) in normal skin, as well as in the treated and control groups as a function of time: on the day of wound making (T0), at 14 days (T1) of the healing process, and after complete wound closure (T2). Means followed by different lowercase and uppercase letters denote by *t*-test, respectively, differences in each group evaluated at T1 and T2 relative to T0, and between groups within each time (T1 and T2).

The immunostained area of COL III in the intact skin (T0) was 3.92 ± 0.61%. At 14 days of wound creation the COL III of the treated group showed a smaller immunostained area (1.96 ± 0.32%) as compared with T0 (*p* < 0.05). However, at 37 days, immunostaining of COL III (3.00 ± 0.58%) was not statistically different in wounds that received LP-PRP relative to T0. Wounds not treated with LP-PRP at 14 days (2.97 ± 0.27%) and 37 days (2.64 ± 0.61%) did not differ from T0. In T1, the area immunostained for COL III in the treated group was smaller (*p* < 0.05) than control. In T2, the values achieved in the immunostained area of the treated animals were higher than in control (Figure 5(a)).

Regarding COL I, the skin of the animals at T0 had an immunostained area of 7.29 ± 0.87%. There was a difference (*p* > 0.05) in staining for COL I at T0 as compared with the other times in animals from the treated group and control. At 14 days after surgical lesion, the immunostained area in control wounds (1.16 ± 0.36%) showed lower values (*p* > 0.05) than the treated group (1.38 ± 0.44%). Nevertheless, at 37 days the treated group had lower values (0.43 ± 0.15%) (*p* > 0.05) than control (0.59 ± 0.15%) (Figure 5(b)). Overall evaluation showed that COL III displayed more intense staining than COL I at all evaluated times.

The extracellular matrix is a complex network of cross-links of proteins and other macromolecules; among them, collagen is the main structural component, essential for the resistance and integrity of all tissues and with a vital role in the healing of wounds [5]. The synthesis of collagen is considered to start from at least the third day after a cutaneous injury. It is known that wounds acquire approximately 20% of their mechanical tensile strength by the end of three weeks from the healing process, which is the period when fibrillar collagens accumulate relatively rapidly. After this period the improvement in resistance slows down, mainly due to the onset of the remodeling of collagens, which start to form large fiber bundles, and also due to the increase in the number of intermolecular crosslink [44], which begins during the remodeling phase.

Lefebvre-Lavoie et al. [45] utilized suppression subtractive hybridization and semiquantitative RT-PCR to identify the gene expression of type I collagen in both healthy skin and at the margin of induced skin wounds in the hemithorax of horses. The evaluation was performed one week after the wound was created, and there was a significant increase in the expression of type I collagen in the samples obtained from the wounds as compared with intact skin in both techniques. Thus, in both physiological conditions and in LP-PRP-treated wounds there is an increase in the expression of type I collagen after one week already, which, according to Schultz et al. [46], corresponds to 80 to 85% of the dermal extracellular matrix.

The results obtained in this study are consistent with those observed by Chamberlain et al. [47], who demonstrated the spatial and temporal location of type I and III collagen immunohistochemically during the healing of wounds induced surgically in horses, healed by second intention. According to the authors, the technique was suitable for the location of the collagens during the assessment period, which was 28 days. The type I collagen remained stable at the beginning of the healing process but reduced dramatically on the 7th day, remaining low until the 28th day. Type III collagen however remained intact in the first hours after the injury, though there was an important increase in its amount 24 h after the surgical procedure, remaining high throughout the entire evaluation period. This collagen accounts for 8 to 11% of the dermal extracellular matrix [46] and should be produced at a larger amount during the final stage of the formation of the extracellular matrix. Subsequently it should be gradually degraded and substituted for type I collagen, which is essential to the increase in the strength of the healing tissue [9, 48], which is reinforced by the collagen crosslink [9].

The intense staining of the papillary and reticular dermis for collagen types I and III, respectively, was expected. The papillary dermis is the loose connective tissue that lies just beneath the epithelium. It typically consists of type III collagen, which forms part of the basal membrane. In contrast, the reticular dermis, which is deeper, is formed by dense connective tissue and is therefore rich in type I collagen, as it provides resistance to the skin [49]. According to Schwartz et al. [7], the increase in type I collagen is progressive, continuing for at least four weeks, which was the maximum period of time in which the authors evaluated this collagen utilizing the Picrosirius Red histochemical technique in wounds of horses located in the thorax and distal region of forelimbs, healed by second intention. In fact, Mignatti et al. [50] mentioned that the elevated collagen synthesis rate within the wound may take from six to 12 months to return to physiological levels, which means that regardless of whether or not the cutaneous injury is treated with PRP, the amount of type I collagen may still be elevated at the moment of wound closure.

The high presence of type III collagen in both groups suggests that the wound was still in the phase characterized by Schultz et al. [30] as contraction and remodeling, although they appeared to be healed macroscopically. This phase is characterized by the reduction in the number of fibroblasts and the balance between production and lysis of collagen. The collagen production must remain high, but with predomination of the functionally oriented collagen fibers over the nonfunctional, and without an increase in the size of the scar [51]. Contrastingly, according to Schultz et al. [46], 40 days after the injury the collagen fibers are not yet organized enough. In this context, the macroscopic examination does not necessarily correspond to the microscopy or the expression of genes of collagen, because the nature of the wound matrix components is modified over time. As a matter of fact, the organization of the collagen in the tissue under repair is changed over the period of several months, and this will slowly increase the tissue tensile strength, which will reach approximately 80% of that corresponding to a normal tissue [5, 29, 46].

Yamauchi and Mechanic [52] define this stage of the healing process after clinical closure as intermediary. According to those authors, although the wound appears to be healed, chemical and structural changes are still in progress. In this stage, the collagen fibrils are grouped and stabilized by the formation of inter- and intramolecular crosslinks. Therefore, this elevated expression of the collagen gene even in this intermediary stage may be because the collagen synthesis still exceeds its extracellular degradation, such that it keeps increasing during scar formation [48]. More specifically, Young and McNaught [29] cite up to two years for the maturation of the scar tissue so that a wound can acquire an epithelium as close as possible to a healthy tissue. In the results presented herein it can be observed that there is a trend towards stabilization in the expression of collagens in the LP-PRP-treated group, which is appropriate. To validate this finding, further research will be necessary to evaluate the gene expression of the collagen at moments (times) subsequent to the clinical healing. Additionally, as mentioned previously, the presence of myofibroblasts at the last evaluation time (T2) used in this study may indicate, as commented by Schwartz et al. [7], that wound healing is still in progress.

Comparing the results obtained in the present study with others conducted with horse skin is practically impossible, because no studies evaluating the expression of the collagens in PRP-treated skin utilizing the immunohistochemical technique have been published. Comparison with studies utilizing other methodologies to evaluate the behavior of the collagens during the healing process is also difficult, because controlled and* in vivo* studies are scarce, and in general they aim to evaluate very precisely a specific time in the different stages of the skin healing process. In addition, most of the experiments have been conducted with cultures of tendon or ligament cells.

To the present date, no research reported results for the immunohistochemical evaluation of collagen types I and III equine cutaneous wounds treated with PRP. Recently, part of the authors who make up the team of the present research study published data on collagen gene expression during different phases of the healing process of PRP-treated skin. However, the technique used for the determination of collagen was qRT-PCR [15]. As observed in the present study, the expression of type III collagen was quite high in the apparently healed wound, whether they were treated or not. Furthermore, regression analysis showed a trend of stabilization in the expression of this collagen in the PRP-treated group, which according to the authors may be a positive aspect of the therapy. Monteiro et al. [13] also evaluated the gene expression of collagen types I and III by PCR in horse's wounds treated with PRP, but in the forelimbs. However this evaluation occurred only after complete healing of the wounds. The authors compared the mean ratio of collagen type I to collagen type III mRNA expression, though they found no difference (*p* > 0.05) between treated and untreated groups in the mean values, which were 0.502 ± 0.155 and 0.542 ± 0.118, respectively.

Although the obtained results cannot be compared with other studies, the presented data are original and valuable for veterinary medicine. Evaluating alterations in the extracellular matrix components is an appropriate form of monitoring tissue repair. Because collagen is the most abundant component of the connective tissue, it is essential during the restoration of the skin function and reflects the quality of the matrix. The experimental trial conducted in distinct moments made it possible to monitor the dynamics of the healing process. The adopted technique, immunohistochemistry, is sensitive, reliable and easily performed, so it is suitable for research on gene expression in different biological components. Moreover, even though few studies have valuable data on density, quantification and/or gene expression of the collagens in the skin of horses, the profile of this expression in the PRP-treated wound should still be better characterized. Obtaining the skin samples for evaluation of extracellular matrix components from different periods after macroscopic healing of the wound may provide new information and verify the efficacy of or inefficacy of the therapy. This corroborates Baksh et al. [53], who mentioned that the benefits of the use of PRP in the “clinical setting remain unclear." According to Brossi et al. [54], despite the fact that the treatment of different tissues has demonstrated “beneficial effects” of platelet-rich component, “clinical evidence of its efficacy remains lacking," although several researchers are conducting quality studies to determine differences between treated and untreated tissues. Furthermore, Romagnoli et al. [55] observed “mild negative correlation between platelet concentration and time to return to sport activity” in spontaneous suspensory ligament injury in their study considering concentrations of platelets in the PRP. Finally, to date, the use of “the therapy in ankle surgery as an orthobiologic does not have an absolute indication” as mentioned by Grambart [56].

Because the main reason for the use of the platelet-rich component is growth factors present in platelets, it is possible that individual variations influence the effectiveness of the therapy. The body region of the horse, the method of obtaining and the composition of PRP (considering the amount of platelets and leukocytes), the presentation form (liquid, gel), route and frequency of application, and the volume and time in which it is administered during the skin healing process are already mentioned as aspects related to the lack of sufficient evidence to confirm the effectiveness of PRP in the treatment of skin wounds. Using the ELISA immunoenzymatic technique, Giraldo et al. [57] have recently reported a significant difference (*p* < 0.001) in the PDGF-BB concentration in PRP gel (P-PRG; activated with 10% calcium gluconate) from Colombian Creole females and young horses (up to 5 years of age) compared with Argentine Creole males and horses over 10.1 years old. Textor et al. [58], however, did not observe differences in the amount of growth factors TGF-*β*1 and PDGF-BB in PRP of males and females. On the other hand, those authors only commented the data obtained for sex evaluation, in precisely four lines at the end of result section, without any other mention in the discussion section.

According to Minimas [59], one of the main features of senile skin is decreased synthesis of collagen. It is possible that the age of the animals used in this study, which was between 16 and 17 years, led to a delay in skin remodeling and thus in the substitution of type III for type I collagen. On the other hand, the similarity in the age of the horses included in the research is a positive aspect, given that several previously published studies used animals of different ages in a way that contributes nothing to the elucidation of the effectiveness or ineffectiveness of PRP. Nevertheless, the contradictory results between studies reinforce the need for further research* in vivo* using homogeneous samples, even considering the age and sex of animals. However, one should be careful in extrapolating and comparing the data obtained* in vitro* with those* in vivo*.

## 4. Conclusions

A single local administration of LP-PRP 12 h after surgical induction of cutaneous injury in the gluteal region of horses results in an important immunohistochemical expression of collagens, particularly COL III, which remains high even during the macroscopic closure of wounds. This greater expression does not result in faster closure of the surgical wound, although microscopically the tissue treated with LP-PRP shows better tissue quality. Additionally, this high COL III staining in both the treated wound and the control wound suggests that the tissue was still weakened, despite being clinically closed.

## Figures and Tables

**Table 1:** Information about manufacturer, code, and dilution of the antibodies utilized in the study.

Primary antibody	Manufacturer	Code	Dilution
Anti-collagen I (mouse monoclonal antibody)	Abcam	Ab90395	1 : 100
Anti-collagen III (rabbit polyclonal antibody)	Abcam	Ab7778	1 : 50
